# Variation of endothelial function during the menstrual cycle evaluated by flow-mediated dilatation of brachial artery

**DOI:** 10.5935/1518-0557.20140022

**Published:** 2014

**Authors:** Augusto H F Brandão, Paula J Serra, Karla Zanolla, Antônio C. V. Cabral, Selmo Geber

**Affiliations:** 1 Department of Obstetrics and Gynecology - Universidade Federal de Minas Gerais - Belo Horizonte - MG - Brazil

**Keywords:** FMD, endothelial function, brachial artery, menstrual cycle

## Abstract

**Objective:**

It is well defined that ovarian steroids play a role in the cardiovascular system, acting as vasoactive compounds. The aim of this study is to assess the endothelial function during the menstrual cycle using flow-mediated dilation of the brachial artery.

**Methods:**

A total of 21 healthy premenopausal women, with regular menstrual cycles, were included in this observational, longitudinal, and prospective study. The endothelium function was assessed by ultrasound during four phases of the menstrual cycle: early follicular phase (EFP), late follicular phase (LFP), early luteal phase (ELP) and late luteal phase (LLP).

**Results:**

We observed a significant variation among the vasodilatation response during the menstrual cycle phases (*P*<0.001). The result was higher during LFP than during ELP (*P*<0.001) or LLP (*P*<0.001). Late luteal phase had higher response than observed during ELP (*P*=0.003) and EFP was higher than LLP (*P*=0.003). There were no significant differences between the results observed during EFP and LFP (*P*=1.0), or EFP and ELP (*P*=0.137).

**Conclusion:**

Our results suggest that the ovarian steroids may play an important role in modulating endothelial function.

## INTRODUCTION

The menstrual cycle is ruled by tightly orchestrated changes in the levels of ovarian estrogen and progesterone, which produce different responses in diverse tissues and organs. It is well defined that ovarian steroids play a role in the cardiovascular system, acting as vasoactive compounds (Hata *et al.*,1990; de Ziegler *et al.*, 1991; Viana *et al.*, 2011). Premenopausal women have smaller risk of cardiovascular disease than postmenopausal women of the same age group (Faustino *et al.*, 2009). Also, it is known that postmenopausal women have a cardiovascular protection after using hormone replacement therapy (HRT), confirmed by an endothelial function improvement (Roh *et al.*, 2004]). Many studies have demonstrated this effect on vascular flow, suggesting that estrogen has a vasodilator effect on arterial vessels, such as central retinal artery (Viana *et al.*, 2011). The actual mechanism is not known but it seems that estrogen promotes vasodilation through endotelial-dependent events stimulating nitric oxide (NO) and prostacyclin production, reducing endothelin and the calcium mediated endothelial independent events (Hayashi *et al.*, 1995).

The flow-mediated dilatation (FMD) of brachial artery is a non-invasive method that measures endothelial function through the vascular response to an induced ischemia. When a sphygmomanometer cuff compresses the arm or forearm, a local tissue ischemia is caused and the response to hypoxia is the NO release, which in turn, causes local arterial vasodilatation (Bots *et al.*, 2005). Therefore we studied the FMD of the brachial artery of 23 healthy premenopausal women to evaluate the endothelial function during the ovulatory menstrual cycle. This dynamic study may increase our understanding of the vascular mechanisms of estrogens and progesterone and also may shed light on the pathogenesis of hot flushes.

## MATERIAL AND METHODS

A total of 23 healthy women with a mean age of 24.76±5.5 years (range from 19-35 years) were evaluated in an observational, longitudinal and prospective study. All participants signed an informed consent form and this study was approved by the local ethics committee (COEP-UFMG). Only women who had regular menstrual cycles (between 25 and 35 days) during the past 12 months and who had menarche at least 5 years before the study were included in the study. Participants were excluded if they were smokers; used vasoactive substances or hormonal drugs within 12 months prior to the study; if there was the presence of hypertension, collagenosis disease, history of ovulatory dysfunction, presence of unbalanced endocrine disease or vascular disease. Body Mass Index (BMI) was calculated by dividing patient’s weight in kilograms by height in meters squared (weight/height^2^). Two women were excluded, as ovulation was not confirmed in the exam cycle.

The same operator performed FMD, at the same time of the day (between 10:00 and 11:00 a.m.) and using the same ultrasound device (Sonoace 8800 - Medson - USA) in order to avoid interobserver and interdevice variations (Mikkonen *et al.*, 1998) and to avoid any possible changes caused by the subject’s circadian cycle (Zaidi *et al.*, 1995). All participants were evaluated in four different phases of the menstrual cycle: early follicular phase (EFP - days 1, 2 or 3), late follicular phase (LFP) (days 12, 13 or 14), early luteal phase (ELP) (days 16, 17 or 18) and late luteal phase (LLP) (25, 26 or 27). These stages were determined according to the previous menstrual cycles’ length as well as by the last menstrual date (LMD), considering that the duration of luteal phase was 14 days. Ovulation was confirmed if serum progesterone, measured on the morning of the 21^st^, 22^nd^, or 23^rd^ day of the menstrual cycle, was >5,000 pg/ml, as previously suggested (Wathen *et al.*, 1984).

Flow-mediated dilatation of the brachial artery measurements were taken with a 7,5 mHz linear transducer color Doppler ultrasound. All women rested for 15 minutes before the examination in supine position. The brachial artery of the dominant arm was identified medially in antecubital fossa. The best image taken of the artery was scanned over a longitudinal section, approximately, 5 cm above the elbow, at the end of the diastole. This moment was monitored using the B-mode of the echocardiographic equipment at the moment that presented the lowest distension of the vessel walls (to prevent larger vascular calibers originated from the vascular distension caused by the systole), which could only be correctly captured by receding the image using the cine loop of the equipment. The arterial diameter was possible to determine by observing still images and calculating the mean of three measurements of the vessel’s caliber (D1). After this first procedure, a pneumatic cuff was inflated, placed on the dominant arm, next to the ultrasound-imaging site, to suprasystolic pressure (250mmHg) for 5 minutes, and after that, the cuff was slowly deflated. One minute after the deflation, the mean of three new measurements of the vessel’s caliber was obtained by the same technique previously described (D2). The FMD value was obtained from the following calculation: FMD (%) = [(D2 - D1)/D1] × 100, where D1 = basal diameter and D2 = post-occlusion diameter. All participants were evaluated in four different phases of the menstrual cycle as described in Study Design.

The vasodilatation response was evaluated for trends over time by repeated measures analyzes of variance with the Hotelling’s T Test. The sphericity of dependent variables was evaluated with Mauchly’s Test (*P*=0.147). The univariate analysis was used to compare differences between two phases employing pairwise comparison. Post hoc test for comparisons of all possible pairs of group of means cycle’s phase was obtained with Bonferroni adjustment. Statistical significance was considered when *P*<0.05. All data are presented as mean values and standard deviation.

## RESULTS

**Figure 1:**
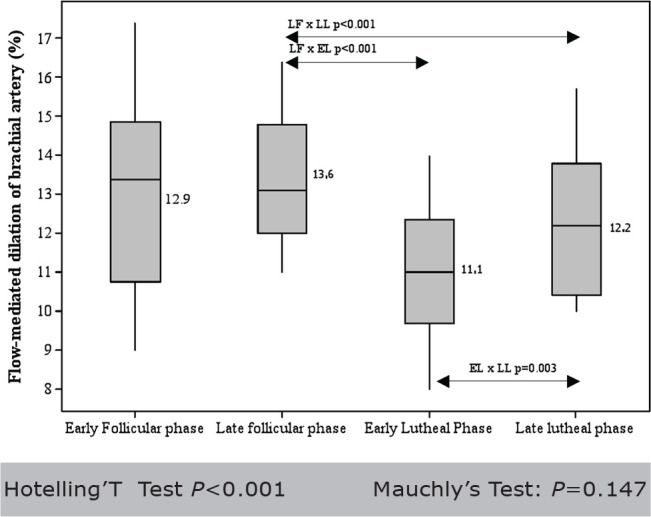
Vascular reactivity study over time period of one menstrual cycle in 21 healthy women

No side effects were observed and none of the participants reported any type of discomfort during or after the exam. All 21 women had the ovulation confirmed by measuring the progesterone serum levels. The mean BMI was 23.34±2.3 (range 17,7 - 27) kg/m^2^ and the mean menstrual cycles’ length was 28.95±1.96 (range 24 - 32) days. The mean baseline brachial artery diameter showed no statistical difference when we compared the four menstrual phases. The mean baseline diameters values of the patients were 0.3 cm in all four phases. The mean variation in Flow-Mediated-Dilatation of brachial artery (FMD in %) during the four phases of menstrual cycle is shown in figure 1 and table 1.

Hotelling’T Test *P*<0.001 Mauchly’s Test: *P*=0.147

**Table 1:** Pairwise comparisons of vascular reactivity study over time period of one menstrual cycle in 21 healthy women by DILA measure

	Mean difference between pairs of menstrual cycles			
FMD %(mean±SD)	Early follicular phase	Late follicular phase	Early luteal phase	Late luteal phase
Early follicular phase 12.93±2.48	...	-0.79 (*P*=1.0)	1.68 (*P*=0.137)	0.57 (*P*=1.0)
Late follicular phase 13.57±0.37	...	...	2.47^a^ (*P*<0.001)	1.36^a^ (*P*<0.001)
Early luteal phase 11.1±1.67	...	...	...	-1.10^a^ (*P*<0.003)
Late luteal phase 12.22±0.39	...	...	...	...

a Bonferroni adjustment

A strong variation was revealed among the vasodilatation response during the menstrual cycle phases (*P*<0.001). The FMD measure was higher during LFP than during ELP (*P*<0.001) or LLP (*P*<0.001). Late luteal phase had higher FMD response than observed during ELP (*P*=0.003) and EFP had a higher FMD response than LLP (*P*=0.003). There were no significant differences between FMD response between EFP and LFP (*P*=1.0), or EFP and ELP (*P*=0.137).

## CONCLUSION

The flow-mediated dilatation was used to evaluate the vascular endothelial function of the brachial artery, as it is a non-invasive and inexpensive technique. A transitory ischemia achieved by a suprasystolic pressure applied in the arm or forearm causes a nitric oxide (NO) release by the healthy vascular endothelium, which makes a compensatory vasodilatation (Munzel, 1984). Therefore this is an important method to assess the endothelial function demonstrated by the endothelium-dependent dilatation, and it can be used to evaluate the effects of the steroid hormones on the arterial vascular bed. Although NO is the most important vasodilator substance released by the endothelium, its short half-life makes this molecule difficult to be assessed through biochemical methods (Al-Qaisi *et al.*, 2008).

All participants were healthy premenopausal women. They all had regular menstrual cycles within the previous 6 months. Ovulation was confirmed by measuring the levels of plasmatic progesterone, as this has been shown to be a non-invasive procedure that provides reliable results (Wathen *et al.*, 1984). We have shown a significant variation among the vasodilatation response during the menstrual cycle phases. This result agrees to what was previously published using Doppler to evaluate the pulsatility index (PI) in different arteries (Hata *et al.*,1990; de Ziegler *et al.*, 1991; Viana *et al.*, 2011; Krejaza *et al.*, 2001]. Hashimoto *et al.* (1995) described similar results during follicular phase but did not observe an antagonizing effect of progesterone on endothelium-dependent vasodilatation. Williams *et al.* (2001) found similar results using FMD to evaluate premenopausal women, however they described larger variations in the FMD values.

During the late follicular phase the FMD was significantly higher than the observed during the early and late luteal phases and similar to the early follicular phase. This can be explained by the fact that is the cycle phase of the higher estradiol level. These results are in agreement with those observed by Hata *et al.* (1990) when studying the PI of the ovarian artery and by Viana *et al.* (2011) when studying the PI of the central retinal arteries, both in women of reproductive age. The vasodilator effect of estrogens was also observed in Doppler studies of other arteries in postmenopausal women (de Ziegler *et al.*, 1991; Faria *et al.*, 2011).

The results of the FMD observed during the early luteal phase were significantly lower than the other phases. These results represent an antagonism on the vasodilatation and can be explained by the higher concentrations of progesterone during this period. This is in agreement with the observed by Williams *et al.* using FMD (Williams *et al.*, 2001). However, Hashimoto *et al.* (1995) also using FMD, did not observe an antagonizing effect of progesterone on endothelium-dependent vasodilatation. Viana *et al.* (2011) when studying the PI of the central retinal arteries of women of reproductive age, during luteal phase, described similar effects of progesterone. Souza *et al.* (2013) also demonstrated that progesterone increases the vascular resistance of ophthalmic and central retinal arteries in climacteric women.

Our results suggest that estrogens are positively correlated in the modulation of endothelial function (vasodilatation effect) and that is antagonized by progesterone, during the ovulatory menstrual cycle. These data obtained using FMD may shed light on the pathology of diseases for which a connection with the sexual steroids has been suggested. Our findings may also be useful to others interested in understanding the vascular dynamics and to researchers running clinical trials, as a physiologic model represents the ideal parameter to be used as reference.

In conclusion, our study demonstrates that the vascular endothelium-dependent response, assessed by FMD, varies during the menstrual cycle. This suggests that estrogen and progesterone might play an important role in the endothelial function modulation in vivo.
